# 

**Published:** 1878-10

**Authors:** 


### CONTENTS.

### COMMUNICATIONS.

Materials for Filling Teeth.—*By Dr. I. P. Wilson*........................ 397
Therapeutical Treatment of Dental Pulps.—*By Dr. A. F. McLain*... 405

### PROCEEDINGS OF SOCIETIES.

Proceedings of the Iowa State Dental Society............................ 410
Proceedings of the Nineteenth Annual Meeting of the Northern Ohio Dental Association.............................................. 421
Wisconsin State Dental Society............................................ 427

### CORRESPONDENCE.

Correspondence..................................................431, 432

### EDITORIAL.

Local Anæsthesia.......................................................... 433
Diamond Disks.............................................................. 433
American Dental Convention.............................................. 434
Virginia State Dental Association Announcement....................... 434
Bibliographical............................................................ 435
Relief for the Dentist..................................................... 435

